# The genome sequence of the Orange-tailed Clearwing, *Synanthedon andrenaeformis *(Laspeyres, 1801)

**DOI:** 10.12688/wellcomeopenres.21110.1

**Published:** 2024-03-20

**Authors:** Douglas Boyes, Peter W.H. Holland, University of Oxford and Wytham Woods Genome Acquisition Lab, Darwin Tree of Life Barcoding collective, Wellcome Sanger Institute Tree of Life Management, Samples and Laboratory team, Wellcome Sanger Institute Scientific Operations: Sequencing Operations, Wellcome Sanger Institute Tree of Life Core Informatics team, Tree of Life Core Informatics collective, Darwin Tree of Life Consortium

**Affiliations:** 1 UK Centre for Ecology & Hydrology, Wallingford, England, UK; 2 University of Oxford, Oxford, England, UK

**Keywords:** Synanthedon andrenaeformis, Orange-tailed Clearwing moth, genome sequence, chromosomal, Lepidoptera

## Abstract

We present a genome assembly from an individual male *Synanthedon andrenaeformis* (the Orange-tailed Clearwing; Arthropoda; Insecta; Lepidoptera; Sesiidae). The genome sequence is 348.4 megabases in span. Most of the assembly is scaffolded into 31 chromosomal pseudomolecules, including the Z sex chromosome. The mitochondrial genome has also been assembled and is 16.65 kilobases in length. Gene annotation of this assembly on Ensembl identified 12,867 protein coding genes.

## Species taxonomy

Eukaryota; Opisthokonta; Metazoa; Eumetazoa; Bilateria; Protostomia; Ecdysozoa; Panarthropoda; Arthropoda; Mandibulata; Pancrustacea; Hexapoda; Insecta; Dicondylia; Pterygota; Neoptera; Endopterygota; Amphiesmenoptera; Lepidoptera; Glossata; Neolepidoptera; Heteroneura; Ditrysia; Apoditrysia; Sesioidea; Sesiidae; Sesiinae; Synanthedonini; *Synanthedon; Synanthedon andrenaeformis* (Laspeyres, 1801) (NCBI:txid1108569).

## Background

The Orange-tailed Clearwing *Synanthedon andrenaeformis* is a diurnal moth in the family Sesiidae found across the western Palaearctic region, particularly in south, central and eastern Europe ( GBIF Secretariat, 2023). The adult moth has a shiny black body marked with delicate yellow bands, and narrow black-edged, otherwise transparent, wings. The common name derives from a fan-shaped ‘anal tuft’ of yellow hair-scales at the terminus of the abdomen.

Like many of the Clearwing moths, the geographical distribution of the species was poorly known until the development of specific pheromone lures, such as the VES lure that attracts males of *S. andrenaeformis* and *S. vespiformis* ( Priesner *et al.*, 1986; Sims *et al.*, 2020; Taylor, 2021). It is now clear that in Britain, *S. andrenaeformis* has a distribution that traces a Z-shape across the south of England: essentially a line running from the Bristol Channel to East Anglia, then southwest to Dorset, and east to Kent. Most of this distribution follows a discontinuous band of chalk and associated scrub and chalk downland habitat ( GBIF Secretariat, 2023; NBN Atlas Partnership, 2023). The wood-boring larvae of *S. andrenaeformis* feed in galleries formed inside the branches of the Wayfaring tree *Viburnum lantana* and the Guelder-rose *Viburnum opulus* ( Rothschild, 1906; South, 1939); both primary food plants are associated with calcareous soils. It has been proposed that dogwood *Cornus sanguinea* may be an alternative food plant ( Sims *et al.*, 2020). Larvae pass through two winters before the adult moths emerge in May to July; a characteristic 3 mm disc-shaped bark cap may be found over the future emergence hole ( Rothschild, 1906).

Here we report a complete genome sequence for the Orange-tailed Clearwing *Synanthedon andrenaeformis* determined as part of the Darwin Tree of Life project. The genome sequence of *S. andrenaeformis* will facilitate research into host-plant specificity and the biology of wood-boring insects, and contribute to the growing set of resources for studying the evolution of Lepidoptera.

## Genome sequence report

**Figure 1.**
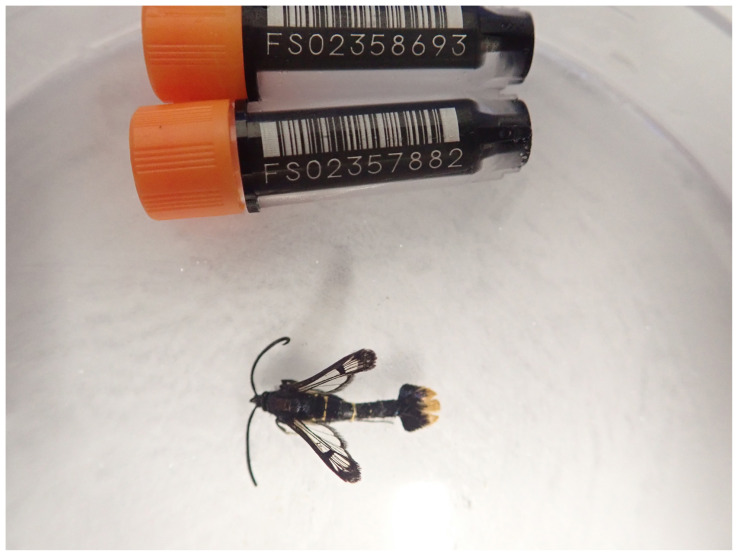
Photograph of the *Synanthedon andrenaeformis* (ilSynAndr1) specimen used for genome sequencing.

The genome was sequenced from a male *Synanthedon andrenaeformis* ( Figure 1) collected from Wytham Woods, Oxfordshire, UK (51.77, –1.34). A total of 106-fold coverage in Pacific Biosciences single-molecule HiFi long reads was generated. Primary assembly contigs were scaffolded with chromosome conformation Hi-C data. Manual assembly curation corrected 8 missing joins or mis-joins, reducing the scaffold number by 2.78%, and increasing the scaffold N50 by 0.55%.

**Figure 2.**
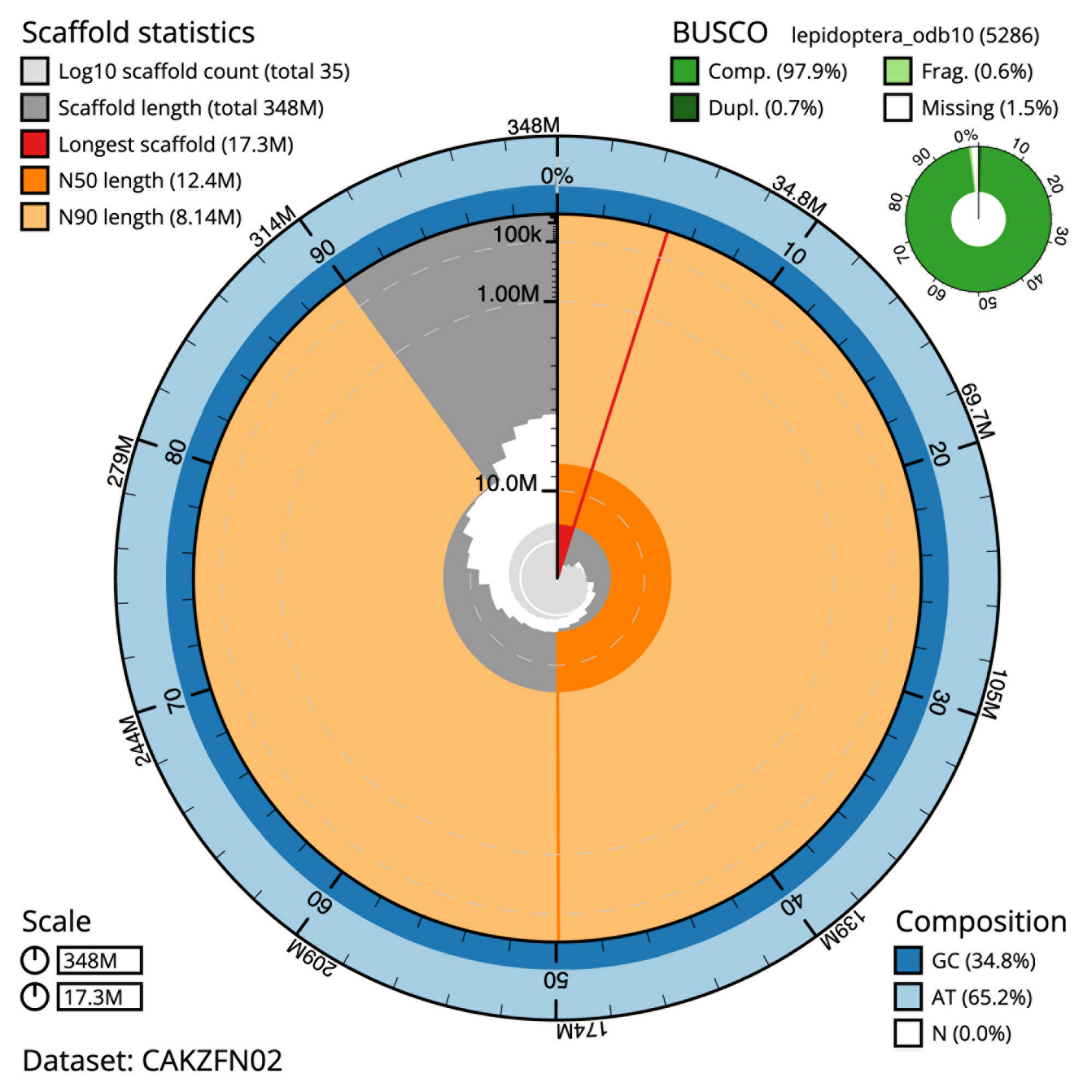
Genome assembly of *Synanthedon andrenaeformis*, ilSynAndr1.2: metrics. The BlobToolKit snail plot shows N50 metrics and BUSCO gene completeness. The main plot is divided into 1,000 size-ordered bins around the circumference with each bin representing 0.1% of the 348,388,281 bp assembly. The distribution of scaffold lengths is shown in dark grey with the plot radius scaled to the longest scaffold present in the assembly (17,284,882 bp, shown in red). Orange and pale-orange arcs show the N50 and N90 scaffold lengths (12,440,847 and 8,142,002 bp), respectively. The pale grey spiral shows the cumulative scaffold count on a log scale with white scale lines showing successive orders of magnitude. The blue and pale-blue area around the outside of the plot shows the distribution of GC, AT and N percentages in the same bins as the inner plot. A summary of complete, fragmented, duplicated and missing BUSCO genes in the lepidoptera_odb10 set is shown in the top right. An interactive version of this figure is available at https://blobtoolkit.genomehubs.org/view/CAKZFN02/dataset/CAKZFN02/snail.

**Figure 3.**
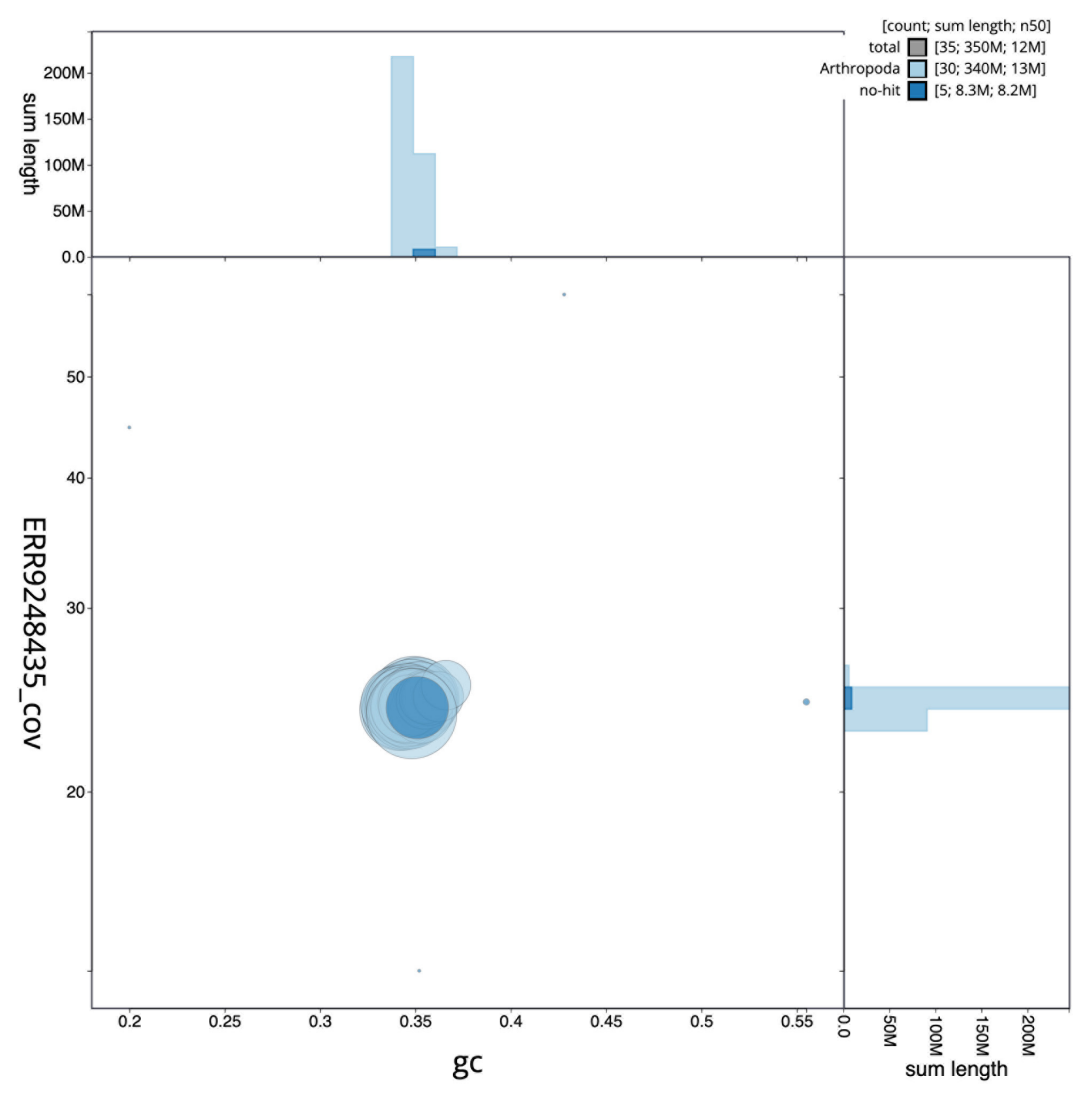
Genome assembly of *Synanthedon andrenaeformis*, ilSynAndr1.2: BlobToolKit GC-coverage plot. Sequences are coloured by phylum. Circles are sized in proportion to sequence length. Histograms show the distribution of sequence length sum along each axis. An interactive version of this figure is available at https://blobtoolkit.genomehubs.org/view/CAKZFN02/dataset/CAKZFN02/blob.

**Figure 4.**
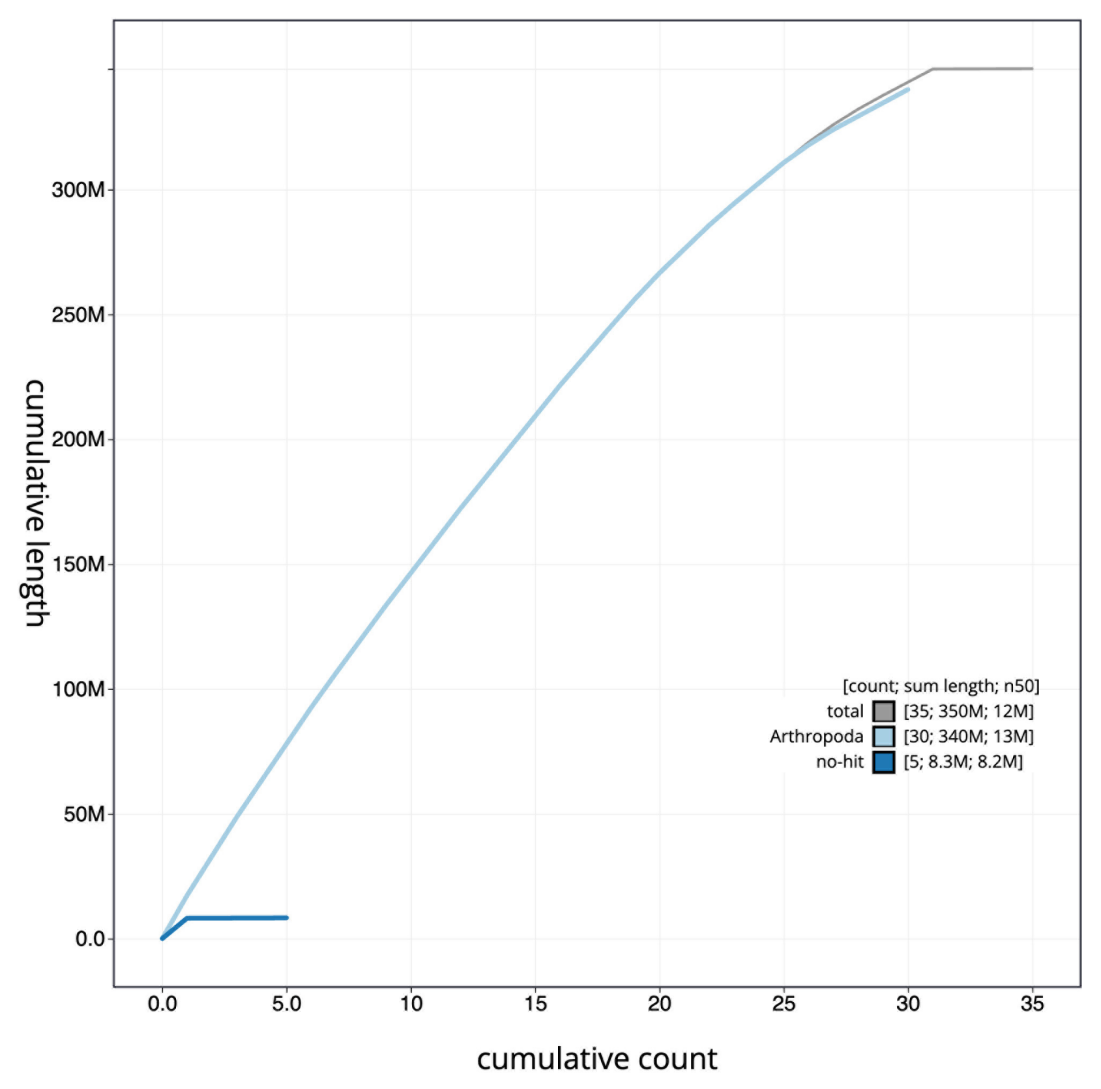
Genome assembly of *Synanthedon andrenaeformis*, ilSynAndr1.2: BlobToolKit cumulative sequence plot. The grey line shows cumulative length for all sequences. Coloured lines show cumulative lengths of sequences assigned to each phylum using the buscogenes taxrule. An interactive version of this figure is available at https://blobtoolkit.genomehubs.org/view/CAKZFN02/dataset/CAKZFN02/cumulative.

**Figure 5.**
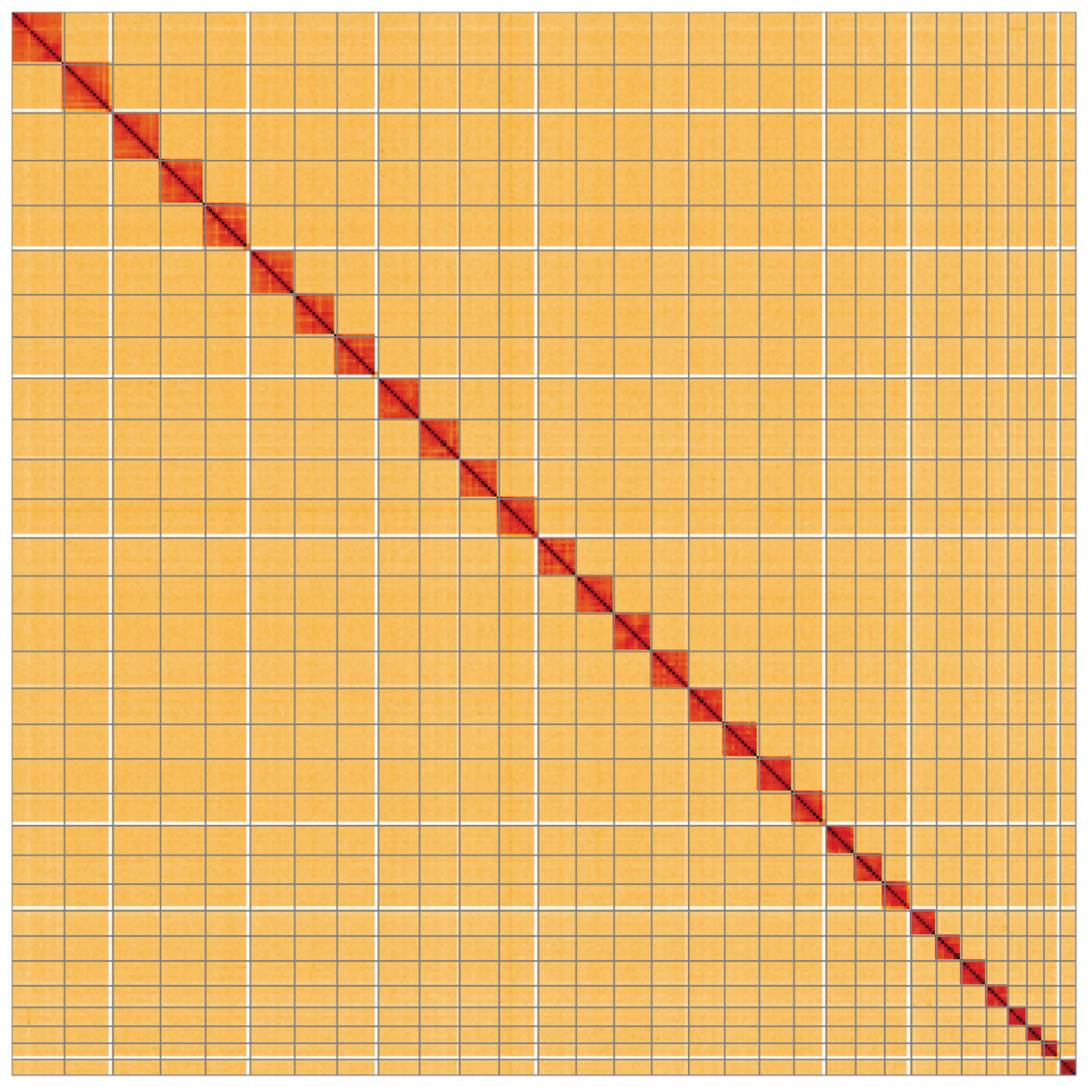
Genome assembly of *Synanthedon andrenaeformis*, ilSynAndr1.2: Hi-C contact map of the ilSynAndr1.2 assembly, visualised using HiGlass. Chromosomes are shown in order of size from left to right and top to bottom. An interactive version of this figure may be viewed at https://genome-note-higlass.tol.sanger.ac.uk/l/?d=bvCHwvS8TbCvV2D-CzlYDw.

The final assembly has a total length of 348.4 Mb in 34 sequence scaffolds with a scaffold N50 of 12.4 Mb ( Table 1). The snail plot in Figure 2 provides a summary of the assembly statistics, while the distribution of assembly scaffolds on GC proportion and coverage is shown in Figure 3. The cumulative assembly plot in Figure 4 shows curves for subsets of scaffolds assigned to different phyla. Most (99.96%) of the assembly sequence was assigned to 31 chromosomal-level scaffolds, representing 30 autosomes and the Z sex chromosome. Chromosome-scale scaffolds confirmed by the Hi-C data are named in order of size ( Figure 5; Table 2). While not fully phased, the assembly deposited is of one haplotype. Contigs corresponding to the second haplotype have also been deposited. The mitochondrial genome was also assembled and can be found as a contig within the multifasta file of the genome submission.

**Table 1.** Genome data for *Synanthedon andrenaeformis*, ilSynAndr1.2.

Project accession data		
Assembly identifier	ilSynAndr1.2	
Species	*Synanthedon andrenaeformis*	
Specimen	ilSynAndr1	
NCBI taxonomy ID	1108569	
BioProject	PRJEB51451	
BioSample ID	SAMEA7701283	
Isolate information	ilSynAndr1, male: abdomen (DNA sequencing); head and thorax (Hi-C sequencing) ilSynAndr2, male: abdomen (RNA sequencing)	
Assembly metrics *		*Benchmark*
Consensus quality (QV)	59.0	*≥ 50*
*k*-mer completeness	100.0%	*≥ 95%*
BUSCO **	C:97.9%[S:97.2%,D:0.7%], F:0.6%,M:1.5%,n:5286	*C ≥ 95%*
Percentage of assembly mapped to chromosomes	99.96%	*≥ 95%*
Sex chromosomes	ZZ	*localised homologous pairs*
Organelles	Mitochondrial genome: 16.65 kb	*complete single alleles*
Raw data accessions		
PacificBiosciences SEQUEL II	ERR9284040, ERR9284041	
Hi-C Illumina	ERR9248438	
PolyA RNA-Seq Illumina	ERR10123688	
Genome assembly		
Assembly accession	GCA_936446665.2	
*Accession of alternate haplotype*	GCA_936447275.2	
Span (Mb)	348.4	
Number of contigs	52	
Contig N50 length (Mb)	10.6	
Number of scaffolds	34	
Scaffold N50 length (Mb)	12.4	
Longest scaffold (Mb)	17.28	
Genome annotation		
Number of protein-coding genes	12,867	
Number of non-coding genes	2,375	
Number of gene transcripts	25,650	

* Assembly metric benchmarks are adapted from column VGP-2020 of “Table 1: Proposed standards and metrics for defining genome assembly quality” from Rhie *et al.* (2021). ** BUSCO scores based on the lepidoptera_odb10 BUSCO set using version 5.3.2. C = complete [S = single copy, D = duplicated], F = fragmented, M = missing, n = number of orthologues in comparison. A full set of BUSCO scores is available at https://blobtoolkit.genomehubs.org/view/CAKZFN02/dataset/CAKZFN02/busco.

**Table 2.** Chromosomal pseudomolecules in the genome assembly of *Synanthedon andrenaeformis*, ilSynAndr1.

INSDC accession	Chromosome	Length (Mb)	GC%
OW387777.2	1	15.87	35.0
OW387778.2	2	15.55	34.5
OW387779.2	3	14.71	35.0
OW387780.2	4	14.66	35.0
OW387781.2	5	14.56	34.5
OW387782.2	6	13.86	34.0
OW387783.2	7	13.51	34.5
OW387784.2	8	13.48	34.5
OW387785.2	9	13.1	34.0
OW387786.2	10	12.94	34.5
OW387787.2	11	12.75	35.0
OW387788.2	12	12.44	34.5
OW387789.2	13	12.37	34.5
OW387790.2	14	12.32	34.5
OW387791.2	15	12.13	35.0
OW387792.2	16	11.77	34.5
OW387793.2	17	11.34	35.0
OW387794.2	18	11.33	34.5
OW387795.2	19	10.56	34.5
OW387796.2	20	9.64	35.0
OW387797.2	21	9.49	35.0
OW387798.2	22	8.69	35.5
OW387799.2	23	8.28	35.5
OW387800.2	24	8.18	35.0
OW387801.2	25	8.14	34.5
OW387802.2	26	7.04	35.5
OW387803.2	27	6.2	35.5
OW387804.2	28	5.53	35.5
OW387805.2	29	5.34	36.0
OW387806.2	30	5.17	36.5
OW387776.2	Z	17.28	35.0
OW387807.2	MT	0.02	20.0

The estimated Quality Value (QV) of the final assembly is 59.0 with *k*-mer completeness of 100.0%, and the assembly has a BUSCO v5.3.2 completeness of 97.9% (single = 97.2%, duplicated = 0.7%), using the lepidoptera_odb10 reference set ( *n* = 5,286).

Metadata for specimens, barcode results, spectra estimates, sequencing runs, contaminants and pre-curation assembly statistics are given at https://links.tol.sanger.ac.uk/species/1108569.

## Genome annotation report

The *Synanthedon andrenaeformis* genome assembly (GCA_936446665.2) was annotated at the European Bioinformatics Institute (EBI) on Ensembl Rapid Release. The resulting annotation includes 25,650 transcribed mRNAs from 12,867 protein-coding and 2,375 non-coding genes ( Table 1; https://rapid.ensembl.org/Synanthedon_andrenaeformis_GCA_936446665.2/Info/Index).

## Methods

### Sample acquisition and nucleic acid extraction

Two male *Synanthedon andrenaeformis* specimens were collected from Wytham Woods, Oxfordshire (biological vice-county Berkshire), UK (latitude 51.77, longitude –1.34) on 2020-06-23 using a VES pheromone lure trap. The specimens were collected and identified by Douglas Boyes (University of Oxford) and then snap frozen on dry ice. The sample used for DNA and Hi-C sequencing had specimen ID Ox000507 (ToLID ilSynAndr1), and the specimen used for RNA sequencing had specimen ID Ox000587 (ToLID ilSynAndr2).

The workflow for high molecular weight (HMW) DNA extraction at the Wellcome Sanger Institute (WSI) includes a sequence of core procedures: sample preparation; sample homogenisation, DNA extraction, fragmentation, and clean-up. In sample preparation, the ilSynAndr1 sample was weighed and dissected on dry ice ( Jay *et al.*, 2023). Tissue from the abdomen was homogenised using a PowerMasher II tissue disruptor ( Denton *et al.*, 2023a). HMW DNA was extracted using the Manual MagAttract v1 protocol ( Strickland *et al.*, 2023b). DNA was sheared into an average fragment size of 12–20 kb in a Megaruptor 3 system with speed setting 30 ( Todorovic *et al.*, 2023). Sheared DNA was purified by solid-phase reversible immobilisation ( Strickland *et al.*, 2023a): in brief, the method employs a 1.8X ratio of AMPure PB beads to sample to eliminate shorter fragments and concentrate the DNA. The concentration of the sheared and purified DNA was assessed using a Nanodrop spectrophotometer and Qubit Fluorometer and Qubit dsDNA High Sensitivity Assay kit. Fragment size distribution was evaluated by running the sample on the FemtoPulse system.

RNA was extracted from abdomen tissue of ilSynAndr2 in the Tree of Life Laboratory at the WSI using the RNA Extraction: Automated MagMax™ *mir*Vana protocol ( do Amaral *et al.*, 2023). The RNA concentration was assessed using a Nanodrop spectrophotometer and a Qubit Fluorometer using the Qubit RNA Broad-Range Assay kit. Analysis of the integrity of the RNA was done using the Agilent RNA 6000 Pico Kit and Eukaryotic Total RNA assay.

Protocols developed by the WSI Tree of Life laboratory are publicly available on protocols.io ( Denton *et al.*, 2023b).

### Sequencing

Pacific Biosciences HiFi circular consensus DNA sequencing libraries were constructed according to the manufacturers’ instructions. Poly(A) RNA-Seq libraries were constructed using the NEB Ultra II RNA Library Prep kit. DNA and RNA sequencing was performed by the Scientific Operations core at the WSI on Pacific Biosciences SEQUEL II (HiFi) and Illumina NovaSeq 6000 (RNA-Seq) instruments. Hi-C data were also generated from head and thorax tissue of ilSynAndr1 using the Arima2 kit and sequenced on the Illumina NovaSeq 6000 instrument.

### Genome assembly, curation and evaluation

Assembly was carried out with Hifiasm ( Cheng *et al.*, 2021) and haplotypic duplication was identified and removed with purge_dups ( Guan *et al.*, 2020). The assembly was then scaffolded with Hi-C data ( Rao *et al.*, 2014) using YaHS ( Zhou *et al.*, 2023). The assembly was checked for contamination and corrected as described previously ( Howe *et al.*, 2021). Manual curation was performed using HiGlass ( Kerpedjiev *et al.*, 2018) and PretextView ( Harry, 2022). The mitochondrial genome was assembled using MitoHiFi ( Uliano-Silva *et al.*, 2023), which runs MitoFinder ( Allio *et al.*, 2020) or MITOS ( Bernt *et al.*, 2013) and uses these annotations to select the final mitochondrial contig and to ensure the general quality of the sequence.

A Hi-C map for the final assembly was produced using bwa-mem2 ( Vasimuddin *et al.*, 2019) in the Cooler file format ( Abdennur & Mirny, 2020). To assess the assembly metrics, the *k*-mer completeness and QV consensus quality values were calculated in Merqury ( Rhie *et al.*, 2020). This work was done using Nextflow ( Di Tommaso *et al.*, 2017) DSL2 pipelines “sanger-tol/readmapping” ( Surana *et al.*, 2023a) and “sanger-tol/genomenote” ( Surana *et al.*, 2023b). The genome was analysed within the BlobToolKit environment ( Challis *et al.*, 2020) and BUSCO scores ( Manni *et al.*, 2021; Simão *et al.*, 2015) were calculated.

Table 3 contains a list of relevant software tool versions and sources.

**Table 3.** Software tools: versions and sources.

Software tool	Version	Source
BlobToolKit	4.1.2	https://github.com/blobtoolkit/blobtoolkit
BUSCO	5.3.2	https://gitlab.com/ezlab/busco
Hifiasm	0.16.1-r375	https://github.com/chhylp123/hifiasm
HiGlass	1.11.6	https://github.com/higlass/higlass
Merqury	MerquryFK	https://github.com/thegenemyers/MERQURY.FK
MitoHiFi	2	https://github.com/marcelauliano/MitoHiFi
PretextView	0.2	https://github.com/wtsi-hpag/PretextView
purge_dups	1.2.3	https://github.com/dfguan/purge_dups
sanger-tol/genomenote	v1.0	https://github.com/sanger-tol/genomenote
sanger-tol/readmapping	1.1.0	https://github.com/sanger-tol/readmapping/tree/1.1.0
YaHS	yahs-1.1.91eebc2	https://github.com/c-zhou/yahs

### Genome annotation

The Ensembl Genebuild annotation system ( Aken *et al.*, 2016) was used to generate annotation for the *Synanthedon andrenaeformis* assembly (GCA_936446665.2) in Ensembl Rapid Release at the EBI. Annotation was created primarily through alignment of transcriptomic data to the genome, with gap filling via protein-to-genome alignments of a select set of proteins from UniProt ( UniProt Consortium, 2019).

### Wellcome Sanger Institute – Legal and Governance

The materials that have contributed to this genome note have been supplied by a Darwin Tree of Life Partner. The submission of materials by a Darwin Tree of Life Partner is subject to the **‘Darwin Tree of Life Project Sampling Code of Practice’**, which can be found in full on the Darwin Tree of Life website here. By agreeing with and signing up to the Sampling Code of Practice, the Darwin Tree of Life Partner agrees they will meet the legal and ethical requirements and standards set out within this document in respect of all samples acquired for, and supplied to, the Darwin Tree of Life Project.

Further, the Wellcome Sanger Institute employs a process whereby due diligence is carried out proportionate to the nature of the materials themselves, and the circumstances under which they have been/are to be collected and provided for use. The purpose of this is to address and mitigate any potential legal and/or ethical implications of receipt and use of the materials as part of the research project, and to ensure that in doing so we align with best practice wherever possible. The overarching areas of consideration are:

•      Ethical review of provenance and sourcing of the material

•      Legality of collection, transfer and use (national and international)

Each transfer of samples is further undertaken according to a Research Collaboration Agreement or Material Transfer Agreement entered into by the Darwin Tree of Life Partner, Genome Research Limited (operating as the Wellcome Sanger Institute), and in some circumstances other Darwin Tree of Life collaborators.

## Data Availability

European Nucleotide Archive: *Synanthedon andrenaeformis* (orange-tailed clearwing). Accession number PRJEB51451; https://identifiers.org/ena.embl/PRJEB51451 ( Wellcome Sanger Institute, 2022). The genome sequence is released openly for reuse. The *Synanthedon andrenaeformis* genome sequencing initiative is part of the Darwin Tree of Life (DToL) project. All raw sequence data and the assembly have been deposited in INSDC databases. Raw data and assembly accession identifiers are reported in Table 1.

## Author information

Members of the University of Oxford and Wytham Woods Genome Acquisition Lab are listed here: https://doi.org/10.5281/zenodo.7125292.

Members of the Darwin Tree of Life Barcoding collective are listed here: https://doi.org/10.5281/zenodo.4893703.

Members of the Wellcome Sanger Institute Tree of Life Management, Samples and Laboratory team are listed here: https://doi.org/10.5281/zenodo.10066175.

Members of Wellcome Sanger Institute Scientific Operations: Sequencing Operations are listed here: https://doi.org/10.5281/zenodo.10043364.

Members of the Wellcome Sanger Institute Tree of Life Core Informatics team are listed here: https://doi.org/10.5281/zenodo.10066637.

Members of the Tree of Life Core Informatics collective are listed here: https://doi.org/10.5281/zenodo.5013541.

Members of the Darwin Tree of Life Consortium are listed here: https://doi.org/10.5281/zenodo.4783558.
