# Supplementary material for: The genome sequence of the Orange-tailed Clearwing, Synanthedon andrenaeformis (Laspeyres, 1801)
Source: Wellcome Open Res. 2024 Mar 20;9:160. [Version 1] doi: 10.12688/wellcomeopenres.21110.1 (PMC11292182; doi:10.12688/wellcomeopenres.21110.1)
Supplement: Peer review history

# Reviewer report: Reviewer response for version 1

**DOI:** 10.21956/wellcomeopenres.23350.r86006

**Published:** 2024-07-31

**Authors:** Annabel Whibley

**Affiliations:** 1 The University of Auckland, Auckland, Auckland, New Zealand; 2 Bragato Research Institute, Lincoln, New Zealand

In this Data Note, Boyes, Holland and colleagues present a reference genome assembly and gene annotation of the Orange-tailed Clearwing ( *Synanthedon andrenaeformis).* The reference is high-quality, using appropriate tools and with comprehensive reporting of the sample collection, data generation and analysis and all associated metadata. Links to data accessions are functional.

**Minor comments:**

 Please preface geographical co-ordinates with latitude and longitude.

  I will continue to query whether this templated detail is correct: “in brief, the method employs a 1.8X ratio of AMPure PB beads to sample to eliminate shorter fragments and concentrate the DNA.”  This ratio of beads is not size-selective,  I believe this should be 0.6x, as in the protocols.io Guidelines.

Are sufficient details of methods and materials provided to allow replication by others?

Yes

Is the rationale for creating the dataset(s) clearly described?

Yes

Are the datasets clearly presented in a useable and accessible format?

Yes

Are the protocols appropriate and is the work technically sound?

Yes

Reviewer Expertise:

Genomics, Bioinformatics, Evolution

I confirm that I have read this submission and believe that I have an appropriate level of expertise to confirm that it is of an acceptable scientific standard.

# Reviewer report: Reviewer response for version 1

**DOI:** 10.21956/wellcomeopenres.23350.r83298

**Published:** 2024-06-07

**Authors:** Gareth Weedall

**Affiliations:** 1 Liverpool John Moores University, Liverpool, England, UK

The data note paper reports the whole genome sequencing, assembly and annotation of the orange-tailed clearwing ( *Synanthedon andrenaeformis*), a diurnal moth with wood-boring larvae.  The rationale for generating the genome assembly, including to provide a platform for further research on host-plant specificity, is clearly presented.  The methodology is, as far as I can judge it, scientifically sound and are clearly described.  The data (project accession PRJEB51451) are publicly accessible. 

 The data note paper reports the whole genome sequencing, assembly and annotation of the orange-tailed clearwing ( *Synanthedon andrenaeformis*), a diurnal moth with wood-boring larvae.  The rationale for generating the genome assembly, including to provide a platform for further research on host-plant specificity, is clearly presented.  The methodology is, as far as I can judge it, scientifically sound and are clearly described.  The data (project accession PRJEB51451) are publicly accessible.

Are sufficient details of methods and materials provided to allow replication by others?

Yes

Is the rationale for creating the dataset(s) clearly described?

Yes

Are the datasets clearly presented in a useable and accessible format?

Yes

Are the protocols appropriate and is the work technically sound?

Yes

Reviewer Expertise:

NA

I confirm that I have read this submission and believe that I have an appropriate level of expertise to confirm that it is of an acceptable scientific standard.
